# NF-κB-responsive miR-155 induces functional impairment of vascular smooth muscle cells by downregulating soluble guanylyl cyclase

**DOI:** 10.1038/s12276-019-0212-8

**Published:** 2019-02-15

**Authors:** Minsik Park, Seunghwan Choi, Suji Kim, Joohwan Kim, Dong-Keon Lee, Wonjin Park, Taesam Kim, Jiwon Jung, Jong Yun Hwang, Moo-Ho Won, Sungwoo Ryoo, Seung Goo Kang, Kwon-Soo Ha, Young-Guen Kwon, Young-Myeong Kim

**Affiliations:** 1 Departments of Molecular and Cellular Biochemistry, Kangwon National University School of Medicine, Chuncheon, Gangwon-do 24341 South Korea; 2 Departments of Obstetrics and Gynecology, Kangwon National University School of Medicine, Chuncheon, Gangwon-do 24341 South Korea; 3 Departments of Neurobiology, Kangwon National University School of Medicine, Chuncheon, Gangwon-do 24341 South Korea; 4 Department of Biology, College of Natural Sciences, Kangwon National University, Chuncheon, Gangwon-do 24341 South Korea; 5 Department of Systems Immunology, College of Biomedical Science, Kangwon National University, Chuncheon, Gangwon-do 24341 South Korea; 6 Department of Biochemistry, College of Life Science and Biotechnology, Yonsei University, Seoul, 03722 South Korea

**Keywords:** RNAi, Cell migration, Disease model

## Abstract

Vascular smooth muscle cells (VSMCs) play an important role in maintaining vascular function. Inflammation-mediated VSMC dysfunction leads to atherosclerotic intimal hyperplasia and preeclamptic hypertension; however, the underlying mechanisms are not clearly understood. We analyzed the expression levels of microRNA-155 (miR-155) in cultured VSMCs, mouse vessels, and clinical specimens and then assessed its role in VSMC function. Treatment with tumor necrosis factor-α (TNF-α) elevated miR-155 biogenesis in cultured VSMCs and vessel segments, which was prevented by NF-κB inhibition. MiR-155 expression was also increased in high-fat diet-fed ApoE^−/−^ mice and in patients with atherosclerosis and preeclampsia. The miR-155 levels were inversely correlated with soluble guanylyl cyclase β1 (sGCβ1) expression and nitric oxide (NO)-dependent cGMP production through targeting the sGCβ1 transcript. TNF-α-induced miR-155 caused VSMC phenotypic switching, which was confirmed by the downregulation of VSMC-specific marker genes, suppression of cell proliferation and migration, alterations in cell morphology, and NO-induced vasorelaxation. These events were mitigated by miR-155 inhibition. Moreover, TNF-α did not cause VSMC phenotypic modulation and limit NO-induced vasodilation in aortic vessels of miR-155^−/−^ mice. These findings suggest that NF-κB-induced miR-155 impairs the VSMC contractile phenotype and NO-mediated vasorelaxation by downregulating sGCβ1 expression. These data suggest that NF-κB-responsive miR-155 is a novel negative regulator of VSMC functions by impairing the sGC/cGMP pathway, which is essential for maintaining the VSMC contractile phenotype and vasorelaxation, offering a new therapeutic target for the treatment of atherosclerosis and preeclampsia.

## Vascular disease: Small RNA as a possible culprit

The overexpression of a microRNA molecule adversely affects the functioning of vascular smooth muscle cells (VSMCs) and may contribute to the development of artherosclerosis and preeclampsia. The interactions between VSMCs and the cells lining blood vessels (endothelium) are crucial for maintaining the healthy phenotype and relaxation of blood vessels. Disruption to these interactions via inflammation, for example, can trigger serious vascular diseases. Young-Myeong Kim at Kangwon National University, Chungcheon, South Korea, and co-workers demonstrated that expression levels of a microRNA-155 are elevated in patients with artherosclerosis and preeclampsia, while an enzyme found in VSMCs called soluble guanylyl cyclase is considerably reduced. Using human and mice tissues, the team showed that miR-155 impairs the contractile phenotype and relaxation of VSMCs by reducing guanylyl cyclase expression. Their findings may inform new therapies for vascular diseases.

## Introduction

The interaction and communication between endothelial cells and vascular smooth muscle cells (VSMCs) play crucial roles in regulating vascular function, such as vascular remodeling and relaxation, through the endothelial nitric oxide synthase (eNOS)-derived NO/soluble guanylyl cyclase (sGC) pathway^1^. In addition to endothelial dysfunction, phenotypic switching of VSMCs from a ‘contractile’ to a pathological ‘synthetic’ state has been shown to play an important role in the pathogenesis of atherosclerosis, as well as in a variety of other major human diseases, including hypertension and preeclampsia^2,3^.

Phenotypic modulation of VSMCs is accelerated by various environmental cues, such as inflammatory cytokines, growth factors, and vascular injury^2^. Additionally, endothelial NO inhibits the proliferation of VSMCs by sGC-dependent cGMP synthesis^4^, indicating that the eNOS/sGC pathway plays a critical role in neointimal proliferation and vascular relaxation during vascular inflammation and following arterial injury. Moreover, eNOS-deficient mice exhibit hypertension and show increased growth of the intima compared to their wild-type counterparts^5,6^. Moreover, sGC expression is downregulated in arterial vessels in spontaneous hypertensive rats^7^. These data suggest that the NO/cGMP pathway is important for VSMC phenotypic modulation and vasorelaxation. Although the expression of sGC is downregulated by inflammatory stimuli, such as TNF-α and IL-1β^8^, the association between sGC downregulation and VSMC dysfunction is poorly understood in inflammatory disease states.

MicroRNAs (miRNAs) are small non-coding RNAs that induce gene silencing through mRNA destabilization and translational repression by complementary binding to the 3′-untranslated region (3′-UTR) of target mRNAs^9^. Growing evidence has shown that several miRNAs are involved in endothelial and VSMC dysfunction, resulting in hypertension, neointimal growth, and vascular remodeling through targeting of specific genes^10–13^. Despite evidence that the sGC/cGMP pathway plays a crucial role in altering or modulating VSMC function, the underlying mechanism involving miRNAs in inflammatory vascular diseases, including atherosclerosis and preeclampsia, remains unclear.

NF-κB-responsive miRNA-155 (miR-155) has been implicated in endothelial dysfunction^14,15^, tumor progression^16^, atherosclerosis^13,17^, and vascular inflammation and permeability^18,19^. However, little is known regarding the involvement of miR-155 in regulating VSMC function and phenotypic switching associated with hypertension and intimal hyperplasia. Here, we found that miR-155 is upregulated in patients with atherosclerosis and preeclampsia and facilitates phenotypic and functional alterations of VSMCs by inhibiting the activity of the sGC/cGMP axis by downregulating sGCβ1 expression, a subunit of dimeric sGC. These findings suggest that TNF-α-induced miR-155 expression is a molecular risk factor for atherosclerotic intima formation and preeclamptic hypertension via impairment of the sGC/cGMP pathway.

## Materials and Methods

Please see the Supplementary Materials in the supplementary information.

### Clinical and animal samples

Animal experiments were performed in accordance with the guidelines of the Institutional Animal Care and Use Ethics Committee of Kangwon National University (KW-171228-1). Because both male and female C57BL/6 ApoE^−/−^ mice are commonly used as animal models of atherosclerosis^20^, we used male C57BL/6 ApoE^−/−^ mice in this study. A total of 12 8-week-old male ApoE^−/−^ mice were randomly divided into two groups (6 in each) and fed either a normal chow diet or a high-fat diet (HFD) containing 0.15% cholesterol and 20% fat for 16 weeks. After mice were sacrificed with CO_2_ gas, their aortas were promptly collected. Human tissue and blood samples were obtained from 11 healthy male adults and 11 male patients with atherosclerosis, as well as 10 healthy pregnant women and 10 patients with preeclampsia according to protocols approved by the Institutional Review Board at Kangwon National University Hospital (KNUH-2017-01-010-004), and informed consent was obtained from all participants. This investigation conformed to the principles outlined in the Declaration of Helsinki.

### Culture of VSMCs and endothelial cells

Human aortic smooth muscle cells (HASMCs) were cultured in SMC medium containing 1% penicillin/streptomycin solution, 1% smooth muscle cell growth supplement and, 2% fetal bovine serum (FBS) at 37 °C in a humidified CO_2_ incubator with 5% CO_2_/95% air. Mouse aortic smooth muscle cells (MASMCs) were isolated from the thoracic and upper parts of the abdominal aorta from 6- to 8-week-old male C57BL6/J mice after an intraperitoneal injection of avertin (250 mg/Kg). Briefly, the stripped aorta was isolated from the sacrificed mouse, cut into 2-mm pieces, treated with type-II collagenase (1 mg/mL) for 1 h to remove endothelial cells, and washed with Dulbecco’s Modified Eagle’s Medium (DMEM) containing 1% penicillin/streptomycin solution, 1.5 ng/mL basic fibroblast growth factor, and 10% FBS. The de-endothelialized aortic pieces were incubated in fresh complete DMEM on gelatin (0.1%)-coated culture dishes for approximately 10 days at 37 °C in a humidified CO_2_ incubator with 5% CO_2_/95% air. MASMCs were identified based on their ‘spindle-shaped’ pattern and further confirmed by double staining using PECAM-1, a specific marker of endothelial cells, and α-smooth muscle actin (α-SMA), a specific marker of SMCs. All cells stained positive for α-SMA, but not PECAM-1. For experiments, VSMCs between passages 3 and 6 were cultured in DMEM containing 0.5% FBS for 24 h and subjected to stimulation with TNF-α (10 ng/mL). Human umbilical vein endothelial cells (HUVECs) were cultured in M199 medium supplemented with a 1% penicillin/streptomycin solution, 1.5 ng/mL basic fibroblast growth factor, and 20% FBS at 37 °C in a humidified CO_2_ incubator with 5% CO_2_/95% air, and only passages 2–6 were used as previously described^21^.

### Transfection with miRNAs and siRNAs

VSMCs and HUVECs were seeded into 6-well plates coated with poly-L-lysine at a density of 2 × 10^5^ cells/well and maintained overnight in SMC medium and M199 medium containing 0.5% FBS, respectively. De-endothelialized aortic rings prepared from male C57BL6/J mice were cultured in DMEM containing 0.5% FBS for 24 h. Cells and aortic rings were transfected with 80 and 100 nM of siRNAs/miRNAs, respectively, in Opti-MEM reduced-serum medium using Lipofectamine RNAiMAX according to the manufacturer’s instructions. Transfected cells and aortic rings were used for further experiments.

### Real-Time Quantitative Polymerase Chain Reaction (qRT-PCR)

Total miRNAs from cells, tissues, and sera were isolated using an miRNeasy Mini kit or miRNeasy serum/plasma kit according to the manufacturer’s instructions. The miR-155 and mRNA levels were measured as previously described^21^. cDNAs were synthesized from 1 μg of miRNAs using a miScript II RT kit. qRT-PCR was performed to determine the miR-155 levels using a miScript SYBR Green PCR Kit as previously described^21^. Total mRNAs were also isolated from cells or tissues using TRIzol reagent, and the mRNA levels of the target genes were determined and quantified by qRT-PCR using their specific primers^22^.

### Western blotting

Cells were suspended in RIPA buffer and incubated on ice for 30 min for complete cell lysis as previously described^21^. Tissue samples were homogenized in ice-cold protein extraction buffer containing 100 mM HEPES (pH 7.9), 10% glycerol, 5% Triton X-100, 250 mM NaF, 5 mM Na_3_VO_4_, and Halt Protease Inhibitor Cocktail (100 × ) using a BioMasher-II homogenizer (Optima, Tokyo, Japan). Cell and tissue lysates were centrifuged at 12,000 *g* at 4 °C for 10 min, and the supernatants were collected to analyze target proteins. The lysates (30 μg protein) were separated by SDS-polyacrylamide gel electrophoresis, and the target protein levels were determined by Western blotting using the appropriate antibodies and chemiluminescent reagents^21^. The relative levels of proteins were quantified by ImageJ software (NIH, Bethesda, MD, USA).

### Measurement of NO and cGMP

The intracellular NO levels were measured *in situ* in endothelial cells using DAF-FM as previously described^21^. HUVECs were treated with TNF-α (10 ng/mL) for 24 h and incubated with 5 μM DAF-FM diacetate for 30 min in a CO_2_ incubator. After washing, the intracellular NO levels were determined from the fluorescence intensity of the DAF-FM/NO adduct by confocal microscopy at excitation/emission wavelengths of 495/515 nm. The level of NO_2_^−^, as a stable oxidized product of NO, was also determined in the culture supernatants via the Griess reaction^23^. Medium alone without cells was used as the negative control. Cells were collected after gentle detachment with 5 mM EDTA, and the cell protein levels were measured according to the Lowry method. To obtain the endothelial cell-derived NO level, the NO_2_^−^ level in media alone was subtracted from the total NO_2_^−^ value. NO_2_^-^ data were expressed as nmoles/mg of cell protein. To evaluate cGMP production, HASMCs (2 × 10^5^ cells) or mouse de-endothelialized aortic rings were transfected with or without miRNAs and stimulated with TNF-α (10 ng/mL for HASMCs and 20 ng/mL for aortic rings) for another 24 h, followed by incubation with or without 100 μM of *S*-nitroso-*N*-acetylpenicillamine (SNAP) or diethylenetriamine diazeniumdiolate (DETA/NO) for 24 h. For co-culture experiments, HASMCs (1 × 10^5^ cells) were transfected with or without miRNAs and co-cultured with HUVECs (1 × 10^5^ cells) in 6-well plates for 24 h. The cGMP concentrations were measured in the aortic ring lysates and conditioned media using an ELISA kit. The protein concentration was determined by a BCA method in total lysates obtained from cells and aortic rings.

### Scratch wound healing assay

HASMCs (2 × 10^4^ cells/well) were cultured in 6-well poly-L-lysine-coated plates using SMC medium containing 0.5% FBS overnight, transfected with miRNAs for 24 h, and stimulated with TNF-α (10 ng/mL) for 24 h. A linear wound was applied to the center of the cell monolayer using a 200-μL pipette tip and treated with or without DETA/NO (100 μM) for 24 h. Images were captured using an Olympus IX71 microscope (Olympus, Tokyo, Japan) equipped with a digital camera. The wound width was calculated as the average distance between the edges of the scratch using ImageJ software.

### Proliferation assay

HASMC proliferation was determined using [^3^H]-thymidine incorporation^14^. Briefly, HASMCs were seeded at a density of 2 × 10^4^ cells per well in poly-L-lysine-coated 6-well plates and cultured in SMC medium containing 0.5% FBS for 24 h. Cells were transfected with miRNAs, stimulated with TNF-α (10 ng/mL) for 24 h, and treated with or without DETA/NO (100 μM) for 24 h, followed by incubation with 1 μCi/mL [^3^H]-thymidine for 6 h. The level of [^3^H]-labeled DNA was determined using a liquid scintillation counter.

### Measurement of sGCβ1 mRNA stability

HASMCs were treated with or without TNF-α (10 ng/mL) in the presence or absence of the NF-κB inhibitor Bay11-7082 (Bay, 5 μM) for 12 h. Cells were further incubated with 5,6-dichloro-1-β-D-ribofuranosylbenzimidazole (40 μg/mL) for the indicated time periods. Total mRNAs were isolated, and the sGCβ1 mRNA levels were determined by qRT-PCR.

### Cytoskeletal organization and morphological analysis

HASMCs were seeded at a density of 1 × 10^3^ cells/well in poly-L-lysine-coated 12-well plates and cultured in SMC medium containing 0.5% FBS for 24 h. Cells were transfected with miRNAs and stimulated with TNF-α (10 ng/mL) for 24 h, followed by treatment with or without DETA/NO (100 μM) for 24 h. The cells were fixed in 3.7% formaldehyde for 30 min at 25 °C, washed gently, and permeabilized with Triton X-100, followed by incubation with Alexa Fluor 488 phalloidin. Nuclei were stained with DAPI. Images were obtained using a confocal laser microscope.

### Mouse aortic vascular tension assay

Because the vasodilatory mechanism can be examined using vascular tissues isolated from mice of either gender^20^, we assessed the effects of TNF-α-induced miR-155 on the vascular tone in aortic vessels isolated from male mice. Briefly, 7-week-old male C57BL/6 J WT (8 animals) and miR-155^−/−^ mice (5 animals) were sacrificed by an intraperitoneal injection of avertin (250 mg/Kg), and the thoracic aortic vessel was rapidly removed. The aorta was placed in ice-cold oxygenated Krebs-Ringer bicarbonate buffer and cleared of adherent connective tissues. The vessels were de-endothelialized by gentle rubbing of the luminal surface and cut into 1.5-mm rings and transfected with miRNAs (100 nM), followed by incubation with TNF-α (20 ng/mL) for 24 h. The vessel strips were suspended between two wire stirrups (150 μm) in a myograph (DMT-620, Aarhus, Denmark) containing 10 mL Krebs-Ringer (pH 7.4, 37 °C) as previously described^14^. The rings were passively stretched at 10-min intervals in increments of 100 mg to reach the optimal tone (600 mg). The responses to the vasoconstrictor phenylephrine or KCl were assessed at different doses (10^−9^–10^−5^ M or 0–0.1 M), and the response to the vasodilator acetylcholine (10^−9^–10^−5^ M) or sodium nitroprusside (SNP, 10^−9^–10^−5^ M) was measured after pre-constriction with a single dose of phenylephrine (10^−5^ M). To further confirm that the vasorelaxation activity occurred in an NO-dependent manner, vessels were treated with the sGC inhibitor 1H-[1,2,4]oxadiazolo[4,3-a]quinoxalin-1-one (ODQ).

### Statistical analysis

Quantitative data are expressed as the mean ± SEM of at least three separate experiments performed in triplicate. Statistical analysis was performed using GraphPad Prism 6 software (GraphPad, Inc., La Jolla, CA, USA). Data that failed the normality and equal variance tests were analyzed by the Mann–Whitney U test for two groups, Kruskal–Wallis or Friedman one-way analysis of variance (ANOVA with Tukey’s post-hoc test, or two-way ANOVA with the Holm–Sidak *post-hoc* test for two independent variables. Significance was established at a *P* value < 0.05.

## Results

### TNF-α-induced miR-155 inhibits sGCβ1 expression

**Fig. 1:**
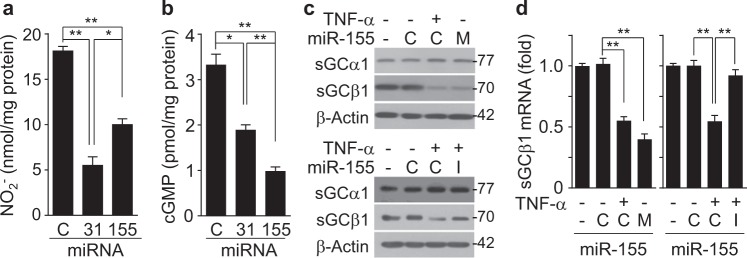
Different effects of TNF-α-induced miR-31 and miR-155 expression on NO and cGMP production in HUVECs. HUVECs were transfected with 80 nM of control miRNA (C), miR-31 mimic (31), or miR-155 mimic (155) and then stimulated with or without TNF-α (10 ng/mL) for 24 h. **a** The NO_2_^-^ levels were determined in culture supernatants by the Griess reaction (*n* = 4). **b** The cellular cGMP levels were determined using a cGMP assay kit (*n* = 4). **c**, **d** HUVECs were transfected with 80 nM of a specific control (C) for miR-155 mimic or miR-155 inhibitor, miR-155 mimic (M), or miR-155 inhibitor (I) and stimulated with or without TNF-α (10 ng/mL) for 24 h. The protein and mRNA levels of sGCα1 and sGCβ1 were measured by Western blotting and qRT-PCR (*n* = 4). *P < 0.05 and **P < 0.01

Although TNF-α induces miR-31 and miR-155 in an NF-κB-dependent manner, which inhibits eNOS expression in cultured HUVECs (Supplementary Figure 1a and b)^14,15^, the autonomous role of these miRNAs in the NO-mediated sGC/cGMP pathway in VSMCs remains unclear. Thus, we first examined the comparative effects of these miRNAs on the eNOS/NO and sGC/cGMP pathways in cultured HUVECs as a model for studying the NO/cGMP axis because endothelial cells express both eNOS and sGC^24^. As anticipated, transfection of HUVECs with miR-31 and miR-155 inhibited NO production; however, the effect of miR-31 was more potent than that of miR-155, as determined by quantification of NO production using the Griess reaction and confocal microscopy (Fig. 1a and Supplementary Figure 1c). Notably, miR-31 exhibited a lower suppressive effect on cGMP production than miR-155 (Fig. 1b). These contradictory findings suggest that miR-155 has silencing activity towards sGC, which consists of the sGCα1 and sGCβ1 subunits. Thus, we examined whether TNF-α-induced miR-155 regulates the expression of these subunits. TNF-α treatment decreased the protein levels of sGCβ1, but not sGCα1, and this decrease was blocked by transfection with a miR-155 inhibitor (Fig. 1c), but not with a miR-31 inhibitor (Supplementary Figure 1b). As anticipated, transfection with a miR-155 mimic inhibited protein expression of sGCβ1, but not sGCα1, as observed for TNF-α (Fig. 1c and Supplementary Figure 1d). A similar inhibitory effect on the mRNA levels of sGCβ1 was detected in HUVECs treated with TNF-α or the miR-155 mimic (Fig. 1d). Because there were no differences in regard to the effects of the miR-155 mimic and inhibitor controls on sGCβ1 expression or cGMP synthesis (Fig. 1c, d), control miRNA was used as a common negative control for both the miR-155 mimic and inhibitor in further experiments. Collectively, these results suggest that miR-155 inhibits the eNOS/cGMP pathway by downregulating the expression of both eNOS and sGCβ1.

### Alterations in miR-155 and sGCβ1 levels in atherosclerotic and preeclamptic disease

**Fig. 2:**
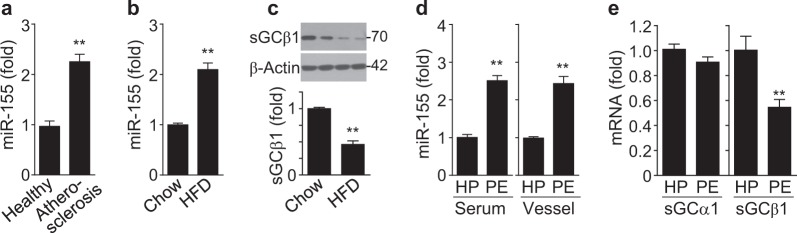
MiR-155 and sGCβ1 are differentially expressed in HFD-fed ApoE^−/−^ mice and atherosclerotic and preeclamptic patients. **a** The miR-155 levels in sera from atherosclerotic patients and healthy controls were assessed by qRT-PCR (*n* = 11 per group). **b**, **c** The levels of miR-155 and sGCβ1 protein were determined in aortic vessels of ApoE^−/−^ mice fed a HFD or a chow diet by qRT-PCR and Western blotting (*n* = 6 per group). **d** The miR-155 levels in sera and placental vessels from healthy pregnant (HP) women and preeclamptic patients (PE) were determined (*n* = 10 per group). **e** The sGCα1 and sGCβ1 mRNA levels in placental vessels from healthy pregnant women and preeclamptic patients were analyzed by RT-PCR (*n* = 10 per group). **P < 0.01

TNF-α is known to be a risk factor of inflammatory vascular disorders, such as atherosclerosis and preeclampsia, which are associated with VSMC dysfunction^3,25^. Therefore, we determined the expression levels of miR-155 and sGCβ1 in these disease states in humans and mice. The miR-155 levels were significantly elevated in sera from patients with atherosclerosis and in aortic vessels from ApoE^−/−^ mice fed a HFD compared to their control counterparts (Fig. 2a, b), whereas the sGCβ1 protein levels were decreased in aortas from HFD-fed mice (Fig. 2c). Additionally, the miR-155 levels were higher in sera and placental vessels from patients with preeclampsia than in those from healthy pregnant women (Fig. 2d), whereas the mRNA levels of sGCβ1, but not sGCα1, were decreased in placental vessels from preeclampsia patients compared to those in healthy controls (Fig. 2e). Therefore, miR-155 biogenesis and vascular sGCβ1 expression are inversely correlated in atherosclerotic and preeclamptic disease.

### TNF-α suppresses sGCβ1 expression via NF-κB-responsive miR-155 biogenesis

**Fig. 3:**
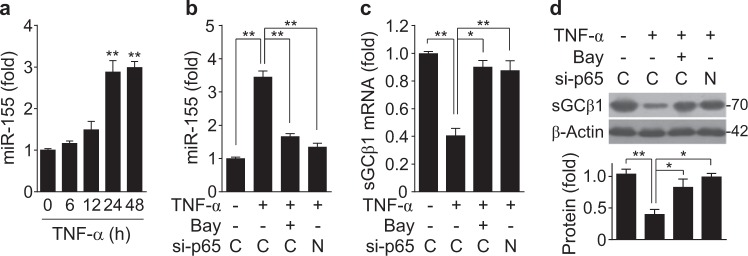
TNF-α inhibits sGCβ1 expression through the biogenesis of NF-κB-responsive miR-155. **a** HASMCs were treated with TNF-α (10 ng/mL), and the miR-155 levels were determined by qRT-PCR (*n* = 3). **b**–**d** HASMCs were transfected with 80 nM of control (C) or NF-κB p65 siRNA (N) and treated with or without TNF-α in the presence or absence of Bay11-7082 (Bay, 5 μM) for 24 h. **b**, **c** The miR-155 and sGCβ1 mRNA levels were determined by qRT-PCR (*n* = 6). **d** The sGCβ1 protein levels were determined by Western blotting (*n* = 3). *P < 0.05 and **P < 0.01

TNF-α is a potent activator of NF-κB and is involved in the pathogenesis of various vascular disorders by impairing the NO/sGC/cGMP pathway^26,27^. We examined whether TNF-α regulates the inverse correlation between miR-155 biogenesis and sGCβ1 expression in HASMCs. Treatment of HASMCs with TNF-α resulted in a significant increase in miR-155 biogenesis in a time-dependent manner (Fig. 3a), and this increase was blocked by treatment with the NF-κB inhibitor Bay11-7082 and siRNA targeting the NF-κB p65 subunit (Fig. 3b), suggesting that NF-κB is essential for miR-155 biogenesis^14,21^. Additionally, TNF-α treatment inhibited the mRNA and protein levels of sGCβ1, but not sGCα1 (Supplementary Figure 2a-c), and this inhibition was reversed by Bay11-7082 and NF-κB p65 siRNA treatment (Fig. 3c, d). Collectively, these data suggest that TNF-α negatively regulates sGCβ1 expression through NF-κB-dependent biogenesis of miR-155.

### sGCβ1 is a target of miR-155

**Fig. 4:**
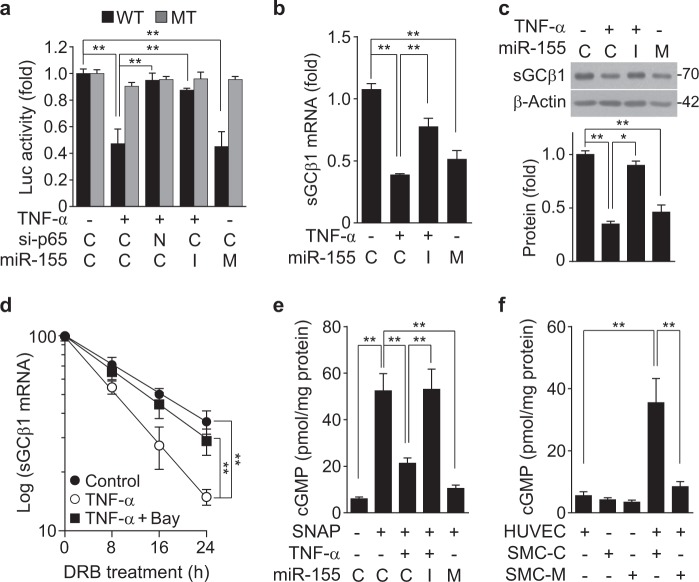
MiR-155 inhibits sGCβ1 expression by targeting the 3′-UTR of its transcript. **a** HASMCs were transfected with psiCHECK-2-sGCβ1 3′-UTR-reporter constructs [wild-type or mutant (MT)] or in combination with 80 nM of control siRNA (C), NF-κB p65 siRNA (N), control miRNA (C), miR-155 mimic (M), or miR-155 inhibitor (I), followed by stimulation with TNF-α for 24 h. Luciferase activity was determined using a dual-luciferase reporter assay kit (*n* = 3). **b**, **c** Transfected HASMCs were stimulated with TNF-α for 24 h. The sGCβ1 mRNA and protein levels were determined by qRT-PCR and Western blotting (*n* = 3). **d** Cells were stimulated with or without TNF-α in the presence or absence of Bay11-7082 (Bay, 5 μM) for 12 h, followed by treatment with 5,6-dichloro-1-β-D-ribofuranosylbenzimidazole (40 μg/mL) for the indicated time periods. The sGCβ1 mRNA levels were determined (*n* = 3). **e** Transfected HASMCs were stimulated with TNF-α for 24 h, followed by further incubation with SNAP (100 μM) for 24 h. The cGMP levels were determined using a cGMP assay kit (*n* = 6). **f** HASMCs were transfected with control miRNA (SMC-C) or miR-155 mimic (SMC-M), followed by co-culture with HUVECs for 24 h. The cGMP levels were determined using an ELISA kit (*n* = 4). *P < 0.05 and **P < 0.01

To examine whether miR-155 inhibits sGCβ1 expression by targeting the 3′-UTR of its mRNA, we analyzed potential miR-155 target sites within the 3′-UTR of sGCβ1 mRNA using TargetScan (targetscan.org). This computational analysis revealed an apparent complementarity between the seed region of miR-155 and the 3′-UTR of human sGCβ1 mRNA, and this sequence is relatively well conserved in other animal species, including non-human primates and mice (Supplementary Figure 3a and b). To confirm this possibility, we examined the role miR-155 in sGCβ1 expression in HASMCs. Transfection with a miR-155 mimic inhibited sGCβ1 expression, and a miR-155 inhibitor reversed the TNF-α-induced decrease in the sGCβ1 protein levels. By contrast, none of the negative controls altered the sGCβ1 protein levels (Supplementary Figure 4a and b), indicating that TNF-induced miR-155 plays an important role in sGCβ1 expression. We next examined whether miR-155 regulates the expression of human sGCβ1 by targeting its 3′-UTR. Treatment with TNF-α resulted in a decrease in the activity of a sGCβ1 mRNA 3′-UTR-based reporter, but not of its mutant reporter, and the decreased wild-type reporter activity was reversed by transfection with NF-κB p65 siRNA or a miR-155 inhibitor (Fig. 4a). Additionally, transfection of a miR-155 mimic inhibited human eNOS mRNA 3′-UTR activity, but not its mutant activity, as did treatment with TNF-α (Fig. 4a). Consistently, TNF-α or miR-155 mimic treatment inhibited the sGCβ1 mRNA and protein levels, and the inhibitory effects of TNF-α were mitigated by treatment with the miR-155 inhibitor (Fig. 4b, c). TNF-α also decreased the half-life of sGCβ1 from 16.2 to 8.5 h, which was significantly restored to 13.8 h by co-treatment with the NF-κB inhibitor Bay 11-7082 (Fig. 4d), suggesting that TNF-α-induced miR-155 destabilizes sGCβ1 mRNA. We next examined the effect of miR-155 on NO-mediated cGMP synthesis. Treatment of HASMCs with TNF-α or a miR-155 mimic inhibited the chemical NO donor SNAP-induced cGMP production compared to untreated control cells, and the inhibitory effect of TNF-α was blocked by treatment with the miR-155 inhibitor (Fig. 4e). We further determined the effect of miR-155 on cGMP production in an in vitro mimic vascular model using a co-culture system of HUVECs and HASMCs because they are able to communicate in the vasculature via eNOS-dependent NO production and sGC-mediated cGMP synthesis, respectively. Co-culture of both cell types resulted in a remarkable increase in cGMP production compared to cultures of each cell type alone, and this increase was significantly decreased only when HASMCs were transfected with the miR-155 mimic (Fig. 4f). These data suggest that TNF-α-induced miR-155 inhibits sGC-dependent cGMP production by targeting the 3′-UTR of sGCβ1 mRNA.

### MiR-155 induces phenotypic switching of VSMCs

**Fig. 5:**
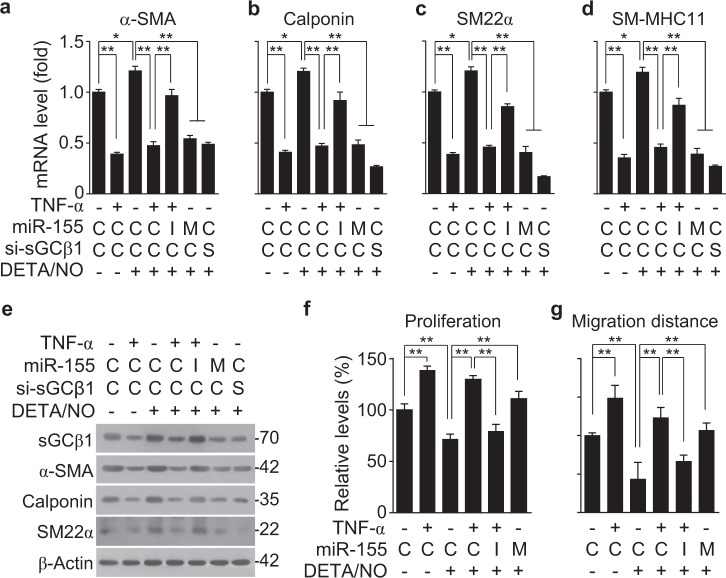
TNF-α-induced miR-155 exerts VSMC phenotypic switching. HASMCs were transfected with 80 nM of control miRNA and siRNA (C), miR-155 mimic (M), miR-155 inhibitor (I), or sGCβ1 siRNA (S) and stimulated with or without TNF-α (10 ng/mL) for 24 h, followed by treatment with DETA/NO (100 μM) for another 24 h. **a**–**e** The mRNA and protein levels of VSMC-specific marker genes were determined by qRT-PCR (*n* = 6) and Western blotting. **f** Cell proliferation was determined by a [^3^H]-thymidine incorporation assay (*n* = 3). **g** The migration distance was calculated as the average distance between the edges of the scratch field (*n* = 5). *P < 0.05, and **P < 0.01

The sGC/cGMP pathway plays an important role in maintaining the VSMC contractile phenotype and vascular relaxation. To examine whether TNF-α-induced miR-155 regulates the expression of contractile phenotype marker genes specific to VSMCs, such as α-smooth muscle actin (α-SMA), smooth muscle calponin, smooth muscle 22α (SM22α), and smooth muscle myosin heavy chain 11 (SM-MHC11), HASMCs were treated with TNF-α, a miR-155 mimic, or a miR-155 inhibitor, and then, the cells were treated with the NO donor DETA/NO as an in vitro model of endothelium-derived NO. Treatment with DETA/NO increased the mRNA levels of the contractile phenotype genes, and their mRNA levels were dramatically decreased by pretreatment with TNF-α, a miR-155 mimic, or sGCβ1 siRNA to approximately 50% or less than those in untreated control cells (Fig. 5a–d). Notably, the suppressive effect of TNF-α on VSMC marker gene expression was reversed by treatment with the miR-155 inhibitor (Fig. 5a–d). Similar expression patterns of the marker proteins were also observed in HASMCs treated with TNF-α, a miR-155 mimic, a miR-155 inhibitor, or sGCβ1 siRNA (Fig. 5e and Supplementary Figure 5a–d). These data suggest that TNF-α-induced miR-155 promotes VSMC phenotypic switching by impairing the sGC/cGMP pathway. We further examined the effect of TNF-α-induced miR-155 on actin rearrangement and cell morphology in HASMCs. Treatment with TNF-α or a miR-155 mimic altered the elongated spindle-shaped morphology of HASMCs, resulting in a spread-out or polygonal/rhomboid cell shape with reduced cytoskeletal rearrangement in the presence of DETA/NO; the TNF-α-induced morphological changes were effectively reversed by treatment with the miR-155 inhibitor (Supplementary Figure 6). We also examined the functional role of TNF-α-induced miR-155 in the proliferation and migration of VSMCs, which are typical characteristics of their synthetic phenotype. TNF-α significantly stimulated HASMC proliferation compared to untreated control cells, whereas DETA/NO had an anti-proliferative effect (Fig. 5f). The TNF-α-induced proliferative effect was not affected by DETA/NO, but was effectively blocked by combined treatment with a miR-155 inhibitor and DETA/NO (Fig. 5f). By contrast, the DETA/NO-induced anti-proliferation effect was suppressed by treatment with a miR-155 mimic, as seen with TNF-α (Fig. 5f). Similar phenomena were also observed for the migration of HASMCs treated with a combination of DETA/NO, TNF-α, a miR-155 mimic, and a miR-155 inhibitor (Fig. 5g and Supplementary Figure 7). Collectively, these data suggest that miR-155 promotes VSMC phenotypic switching from the contractile to the synthetic state by inhibiting the NO/sGC/cGMP pathway under inflammatory conditions.

### TNF-α-responsive miR-155 impairs NO-mediated vasorelaxation

**Fig. 6:**
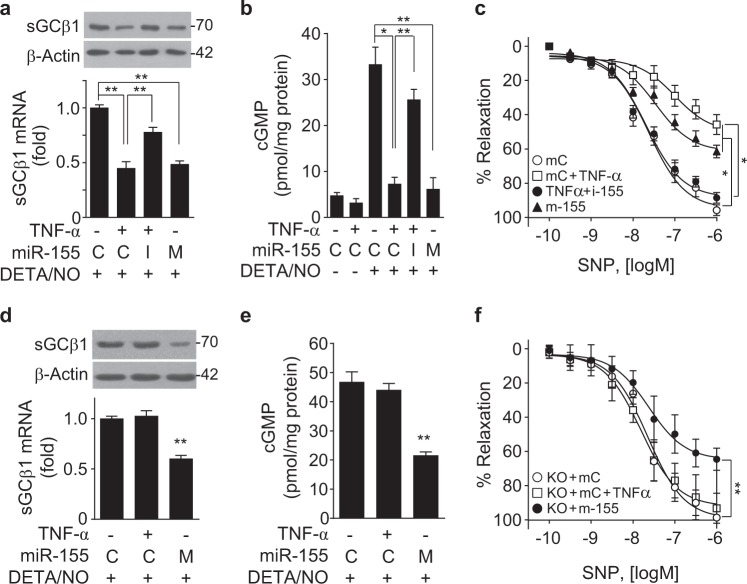
MiR-155 suppresses vasorelaxation by inhibiting the sGC/cGMP pathway. **a** Mouse de-endothelialized aortic rings were transfected with 100 nM of control miRNA (C), miR-155 mimic (M), or miR-155 inhibitor (I), followed by treatment with or without TNF-α (20 ng/mL) for 24 h. The sGCβ1 mRNA and protein levels were determined by qRT-PCR (*n* = 3) and Western blotting. **b** De-endothelialized aortic rings were transfected with miR-155 mimic and miR-155 inhibitor and stimulated with or without TNF-α for 24 h, followed by treatment with DETA/NO for another 24 h. The cGMP levels were determined in vessel lysates using a cGMP assay kit (*n* = 3). **c** De-endothelialized aortic rings were transfected with control miRNA, miR-155 mimic (m-155), or miR-155 inhibitor (i-155), followed by treatment with TNF-α for 24 h. The cumulative vascular relaxation response of the aortic rings to SNP was measured by myography (*n* = 8). **d**–**f** De-endothelialized aortic rings from miR-155^−/−^ (KO) mice were transfected with 100 nM of control miRNA (C, mC) or miR-155 mimic (M, m-155) and stimulated with or without TNF-α for 24 h, followed by treatment with DETA/NO (100 μM) for another 24 h. **d** The sGCβ1 mRNA levels were determined (*n* = 5). **e** The cGMP levels were determined using a cGMP assay kit (*n* = 5). **f** The relaxation response of de-endothelialized miR-155^−/−^ (KO) aortic rings to SNP was measured by myography (*n* = 5). *P < 0.05 and **P < 0.01

We next examined whether TNF-α-induced miR-155 regulates NO-mediated vasorelaxation in mouse aortic rings. Treatment with TNF-α or a miR-155 mimic decreased the sGCβ1 mRNA and protein levels in de-endothelialized mouse aortic vessels, and the effects of TNF-α were mitigated by treatment with the miR-155 inhibitor (Fig. 6a). Consequently, pretreatment with TNF-α or a miR-155 mimic significantly inhibited DETA/NO-induced cGMP production in vessel lysates and media of *ex vivo* cultured de-endothelialized mouse vessels, and the inhibitory effect of TNF-α was mitigated by treatment with the miR-155 inhibitor (Fig. 6b and Supplementary Figure 8a). Treatment of de-endothelialized vessels with TNF-α, a miR-155 mimic, or a miR-155 inhibitor did not alter the vasoconstrictor responses to phenylephrine and KCl (Supplementary Figure 8b and c), suggesting that VSMCs in de-endothelialized vessels maintained their normal function and integrity. In addition, these treatments did not induce a vasodilatory response of de-endothelialized vessels to the endothelium-dependent vasodilator acetylcholine compared to the vasorelaxation activity of intact vessels (Supplementary Figure 8d), suggesting that TNF-α-induced miR-155 does not affect vascular relaxation in the absence of an NO-generating source. Notably, treatment of de-endothelialized vessels with TNF-α or a miR-155 mimic significantly inhibited the vasorelaxant response to the chemical NO donor sodium nitroprusside (SNP), although the inhibitory effect of TNF-α was reversed by the miR-155 inhibitor (Fig. 6c). As expected, the vasorelaxant response to the sGC activator YC-1 was inhibited by treatment with TNF-α and a miR-155 mimic, and the inhibitory effect of TNF-α disappeared following treatment with a miR-155 inhibitor (Supplementary Figure 8e). These data suggest that TNF-α-induced miR-155 impairs NO-dependent vasorelaxation by post-transcriptional downregulation of sGCβ1 expression in VSMCs.

### TNF-α does not regulate NO-mediated VSMC function in miR-155^−/−^ aortic vessels

We examined whether TNF-α regulates the sGC/cGMP axis and VSMC function in de-endothelialized aortic vessels from miR-155^−/−^ mice. TNF-α did not regulate sGCβ1 expression or cGMP production in miR-155^−/−^ aortic vessels exposed to DETA/NO compared to those in untreated vessels from miR-155^−/−^ mice, whereas transfection with a miR-155 mimic decreased sGCβ1 expression and cGMP production in miR-155^−/−^ aortic vessels (Fig. 6d, e). Consistently, TNF-α did not alter the mRNA levels of VSMC-specific marker genes, such as α-SMA, calponin, SM22α, and SM-MHC11, in de-endothelialized miR-155^−/−^ aortic rings, while transfection of a miR-155 mimic downregulated the levels of these marker genes (Supplementary Figure 9a-d). De-endothelialized vessels from wild-type and miR-155^−/−^ mice showed similar responses to KCl-induced vasoconstriction (Supplementary Figure 10); however, treatment of de-endothelialized miR-155^−/−^ vessels with a miR-155 mimic, but not TNF-α, inhibited SNP-induced vasodilation (Fig. 6f). These data suggest that miR-155 inhibits the sGC/cGMP pathway, which is essential for maintaining the VSMC phenotype and vasodilation.

## Discussion

VSMCs are highly specialized cells that have a contractile capability in blood vessels and, in concert with endothelial cells, are essential for the maintenance of vascular function. Through constriction and relaxation, they control the luminal diameter, which enables blood vessels to maintain a normal blood pressure. VSMCs possess phenotypic plasticity that allows for rapid adaptation to fluctuating environmental cues, such as growth factors, mechanical influences, and various inflammatory mediators. These conditions stimulate phenotypic changes of VSMCs from a quiescent contractile phenotype to an active synthetic state, which results in intimal hyperplasia and impaired contractility. Although inflammatory responses stimulate VSMC phenotypic modulation, the underlying mechanism is largely unknown.

The present study shows that TNF-α stimulates NF-κB-dependent biogenesis of miR-155, the levels of which were substantially upregulated in both mouse atherosclerotic arteries and samples from patients with atherosclerosis and preeclampsia. The miR-155 levels were inversely correlated with sGCβ1 expression in atherosclerotic mice and preeclamptic patients. We also found that TNF-α and miR-155 mimic treatment induced VSMC phenotypic switching and mitigated NO-induced vasodilation by suppressing sGC-dependent cGMP production. However, treatment with a miR-155 mimic, but not TNF-α, dysregulated VSMC function in miR-155^−/−^ aortic vessels. Therefore, these results suggest that NF-κB-responsive miR-155 is a novel negative regulator of VSMC function (Supplementary Figure 11), which plays important roles in the induction of intimal hyperplasia and hypertension associated with atherosclerosis and preeclampsia.

Inflammatory cytokines, such as TNF-α and IL-6 that are known to be potent activators of NF-κB, are risk factors for vascular disorders, including atherosclerosis, preeclampsia, and hypertension^28–30^, which suggests that inflammatory cytokines stimulate phenotypic modulation of VSMCs by downregulating the expression of their contractile biomarker genes, likely through activation of NF-κB^31^. In fact, inhibition of NF-κB activation using a pharmacological inhibitor prevents lipopolysaccharide-induced VSMC proliferation and atherosclerosis lesions in ApoE^−/−^ mice^32^. Additionally, SMC-selective inhibition of NF-κB has been shown to attenuate SMC phenotypic switching and neointima formation following vascular injury^31^. These data suggest that NF-κB plays an important role in repressing VSMC differentiation marker genes. Although NF-κB stimulates transcriptional expression of numerous genes, our data demonstrated that TNF-α-induced activation of NF-κB inhibited sGCβ1 expression through the biogenesis of miR-155, which directly targets the 3′-UTR of its transcript. Therefore, NF-κB-responsive miR-155 is a negative regulator of the sGC/cGMP pathway and is responsible for maintaining the VSMC contractile phenotype.

In the vasculature, eNOS-derived NO in the endothelium diffuses to the underlying vascular smooth muscle where it acts to maintain vascular tone and VSMC plasticity by stimulating the sGC/cGMP pathway, with subsequent activation of cGMP-dependent protein kinase (PKG). sGC expression has been shown to be downregulated in spontaneously hypertensive rats and angiotensin II-induced hypertensive mice^33–35^, and whole-body or SMC-specific deletion of sGCβ1 causes hypertension in mice^36,37^. These observations indicate that impairments of the sGC/cGMP pathway contribute to the pathogenesis of hypertension. In addition, pharmacological activation of sGC has been shown to exert an anti-atherosclerotic effect in ApoE^−/−^ mice^38^, as well as inhibit VSMC proliferation and arterial neointima formation in a rat model of experimental balloon injury^39^. However, there are contradictory data showing that genetic deletion of sGCα1, another subunit of dimeric sGC, in mice prevents the phenotypic switching and proliferation of VSMCs^40^. Based on these observations, it appears that the sGC/cGMP pathway contributes to VSMC dysfunction associated with intimal growth and hypertension. Here, we also found that miR-155 induced VSMC phenotypic switching and inhibited NO-mediated vasorelaxation by downregulating sGCβ1 expression. These results suggest that NF-κB-responsive miR-155 disrupts the maintenance of VSMC function, leading to intimal proliferation and vasoconstriction.

Among the many different intracellular messengers, cGMP is largely generated in SMCs by NO-mediated activation of sGC. cGMP exerts its anti-proliferative and vasodilatory effects through activation of PKG^41,42^. Activated PKG phosphorylates the vasodilator-stimulated phosphoprotein at Ser239^42^, resulting in actin polymerization and maintenance of the VSMC contractile phenotype. In addition, the cGMP/PKG pathway induces vasorelaxation via activation of myosin light chain phosphatase in VSMCs^43^. Indeed, genetic deletion of PKG in mice results in impaired vasorelaxation and hypertension^44^, whereas expression of a constitutively active PKG reduces the neointima formation associated with the phenotypic modulation of VSMCs following vascular injury^45^. Thus, the sGC/cGMP/PKG pathway is a crucial signaling component in VSMC-dependent vascular function and homeostasis. Our data show that NF-κB-induced miR-155 downregulates sGCβ1 expression in VSMCs, leading to inhibition of the NO/cGMP pathway and VSMC dysfunction under inflammatory conditions.

The sGC/cGMP axis is activated in the vasculature by endothelium-derived NO production. Thus, eNOS-deficient mice induce neointimal proliferation after vascular injury and spontaneous hypertension^5,6,46^, indicating that eNOS-derived NO plays an important role in preventing VSMC proliferation (hyperplastic remodeling) and hypertension. Although eNOS is constitutively expressed, recent studies have reported that this gene is negatively regulated by the NF-κB-dependent biogenesis of miR-155 in humans, but not in mice, leading to endothelial dysfunction and vasoconstriction^14,15^. In addition, our previous data have shown that miR-155 also downregulates PKG1 expression^47^. Moreover, in the present study, we demonstrate that miR-155 potentially inhibits sGCβ1 expression in both humans and mice. These findings suggest that TNF-α-induced miR-155 is crucially involved in vascular dysfunction by targeting the mRNA transcripts of eNOS, sGCβ1, and PKG1 in humans. These data indicate that NF-κB plays a key role in the pathogenesis of preeclampsia, atherosclerosis, and other inflammatory vascular disorders through the biogenesis of miR-155.

Although at first glance atherosclerosis and preeclampsia seem to be caused by independent risk factors, both syndromes can, in fact, be caused by many common risk factors^48^, including inflammatory cytokines and oxidative stress, and the underlying mechanism(s) associated with preeclampsia is linked to a large contribution from cardiovascular risk factors that are present prior to gestational hypertension^49^. Indeed, women with preeclampsia develop atherosclerosis-like lesions in the arteries of their uterine wall during pregnancy and have a two-fold higher risk for subsequent cardiovascular disease, suggesting shared underlying mechanisms in the pathogenesis of both diseases^50^. There are common clinical and cellular features of inflammation, NF-κB activation, and endothelial and VSMC dysfunction in the pathogenesis of atherosclerosis and preeclampsia^4,14,28,35,48^. Several studies have shown that NF-κB-responsive miR-155 causes dysfunction in both endothelial cells and VSMCs by downregulating eNOS expression^14,15,51^. Additionally, the present study showed that TNF-α-induced miR-155 promoted VSMC proliferation and inhibited vasorelaxation in an NF-κB-dependent manner by downregulating the expression of sGCβ1. These events are crucial for the pathogenesis of atherosclerosis and preeclampsia, both of which are associated with impaired VSMC dysfunction^49^.

Taken together, the present study demonstrates that TNF-α-induced miR-155 inhibits sGCβ1 expression in an NF-κB-dependent manner by targeting its transcript, resulting in inhibition of the sGC/cGMP pathway. The dysfunctional sGC/cGMP axis results in phenotypic alterations of VSMCs and impairs vascular relaxation, both of which are associated with various vascular diseases. These findings offer a possible mechanistic link between NF-κB and VSMC dysfunction through miR-155-mediated downregulation of sGCβ1 during the development of atherosclerosis and preeclampsia. In combination with our previous studies^14,15,47^, the present data support that miR-155, a negative regulator of eNOS, sGCβ1, and PKG1 in the vasculature, is a common therapeutic target for the development of treatments for atherosclerosis and preeclampsia.

## Supplementary information

Supplemental Materials

Supplemental Figures
