# Supplementary material for: NF-κB-responsive miR-155 induces functional impairment of vascular smooth muscle cells by downregulating soluble guanylyl cyclase
Source: Exp Mol Med. 2019 Feb 15;51(2):17. doi: 10.1038/s12276-019-0212-8 (PMC6376011; doi:10.1038/s12276-019-0212-8)
Supplement: Supplementary file 1 — Supplemental Materials [file 12276_2019_212_MOESM1_ESM.pdf]

SUPPLEMENTAL MATERIAL

Mice

Strain	Source	Background Strain	Sex	Number
miR-155$^{-/-}$	Jackson Laboratory	129S6/SvEvTac × C57BL/6NTac mixed background	M	8
Wild type	Orient Biotech	C57BL/6	M	48

Media

Media	Source	Catalog #
Basic FGF	Merck Millipore	01-106
Smooth muscle cell medium	ScienCell	1101
Smooth muscle cell growth supplement	ScienCell	1152
Fetal bovine serum	ScienCell	0010
Penicillin/streptomycin solution	ScienCell	0503
Medium 199/EBSS	HyClone	SH30253.01
DMEM/High glucose	HyClone	SH30243.01
Fetal bovine serum	HyClone	SH30919.03
Penicillin-streptomycin solution	HyClone	SV30010

Antibodies

Antigen	Source	Catalog #	Working concentration	Species
eNOS	BD Bioscience	610297	1:1000	Mouse
SM22α	Abcam	ab14106	1:5000	Rabbit
Calponin	Abcam	ab46794	1:5000	Rabbit
α-SMA	Santa Cruz	sc-130616	1:1000	Mouse
sGCα1	Cayman Chemical	160895	1:1000	Rabbit
sGCβ1	Cayman Chemical	160897	1:1000	Rabbit
CD31	BD Bioscience	550274	1:200	Rat
2nd antibodies	Thermo Fisher Scientific	31340/31460	1:3000	Goat

Cultured Cells

Name	Source	Sex
Human aortic smooth muscle cells	ScienCell	Unknown
Human umbilical vein endothelial cells	Isolation from human umbilical cords	F
Mouse aortic smooth muscle cells	Isolation from aortas of C57BL/6	M

qRT-PCR primers

Gene	Sense	Anti-sense	Reference
hsGCα1	AAATCAATGTCAGCCCAACA	AAACACGAAACCAGGACAGTC	1
hsGCβ1	GCCAGGTTCAAGTAGATGGTG	GGCATCCGCTGTCCTATG	1
α-SMA	GATCTGGCACCACTCTTTCTAC	CAGGCAACTCGTAACTCTTCTC	2
Calponin	ATGTCCTCTGCTCACTTCAAC	CACGTTCACCTTGTTTCCTTTC	2
SM-MHC11	GGCGAACCTAGACAAGAATAAG	CTGGATGTTGAGAGTGGAGATG	2
SM22α	TCCAGACTGTTGACCTCTTTG	TCTTATGCTCCTGCGCTTTC	2
hGAPDH	GGGGCTCTCCAGAACATCAT	GGTCAGGTCCACCACTGACA	2
msGCβ1	GCGGTACTCTTGCCTGGAAG	GACCATAATTGCGGATCACCA	3
mGAPDH	ACCATCTTCCAGGAGCGAGAC	GCCTCCTCCATGGTGGTGAA	3

Kits

Kit	Source	Catalog #
Parameter cGMP	R&D Systems	KGE003
miRNeasy Mini Kit	Qiagen	217004
miScript SYBR Green PCR Kit	Qiagen	218073
Luciferase reporter assay	Promega	E1960

siRNAs and miRNAs

siRNA/miRNA	Source	Catalog #	Working concentration
miR-31 mimic	Qiagen	MSY0000089	Cell : 80 nM
miR-31 inhibitor	Qiagen	MIN0000089	Cell : 80 nM
miR-155 mimic	Qiagen	MSY0000646	Cell : 80 nM<br>Vessel : 100 nM
miR-155 inhibitor	Qiagen	MIN0000646	Cell : 80 nM<br>Vessel : 100 nM
Allstars negative control siRNA	Qiagen	1027281	Cell : 80 nM<br>Vessel : 100 nM
miScript inhibitor negative control	Qiagen	1027271	Cell : 80 nM<br>Vessel : 100 nM
NF-κB p65 siRNA	Santa Cruz	sc-29410	80 nM
Control siRNA	Santa Cruz	sc-37007	80 nM
sGCβ1 siRNA	Bioneer	1066-497/499/500	80 nM
AccuTarget negative control siRNA	Bioneer	SN-1001-CFG	80 nM
Lipofectamine RNAiMAX	Invitrogen	56532	Cell : 3 μl/ml<br>Vessel : 5 μl/ml

Chemicals

Chemical	Source	Catalog #	Working concentration
S-nitroso-N-acetylpenicillamine	Cayman Chemical	67776-06-1	100 μM
Diethylenetriamine diazeniumdiolate	Cayman Chemical	146724-94-9	100 μM
DAF-FM diacetate	Invitrogen	D23844	5 μM
Sodium nitroprusside	Sigma-Aldrich	1614501	10^{-10}-10^{-6} M
Acetylcholine chloride	Sigma-Aldrich	A6625	10^{-9}-10^{-5} M
Phenylephrine	Sigma-Aldrich	P1240000	10^{-9}-10^{-5} M
Alexa Fluor 488 phalloidin	Thermo Fisher Scientific	A12379	0.5 U/mL
DAPI	Sigma-Aldrich	D9542	1 μg/mL
ODQ	Sigma-Aldrich	O3636	1 mM
TNF-α	R&D Systems	210-TA	10 ng/mL
Bay11-7082	Calbiochem	196870	5 μM
Avertin	Sigma-Aldrich	T48402	250 mg/kg
Halt protease inhibitor cocktail	Thermo Fisher Scientific	78425	10 μL/mL
5,6-Dichlorobenzimidazole 1-β-D-ribofuranoside	Sigma Aldrich	D1916	20 μg/mL
Poly-L-Lysine	ScienCell	0403	1 μg/mL
Type II collagenase	Worthington	LS004177	1 mg/mL
Gelatin	Sigma-Aldrich	G2500	0.1 %
Formaldehyde solution	Junsei	69360S0380	3.7 %
Triton X-100	Sigma-Aldrich	X100	5 %
HEPES	Duchefa Biochemie	H1504	100 mM
Glycerol	Sigma-Aldrich	G6279	10 %
NaF	Sigma-Aldrich	S7920	250 mM
Na_3VO_4	Sigma-Aldrich	S6508	5 mM
YC-1	Sigma-Aldrich	Y102	10^{-8}-10^{-4} M

References

1. Wen H.C. *et al.* Elevation of soluble guanylate cyclase suppresses proliferation and survival of human breast cancer cells. *PLoS One.* **10**, e0125518 (2015).

2. Yang L. *et al.* Lactate promotes synthetic phenotype in vascular smooth muscle cells. *Circ. Res.* **121**, 1251-1262 (2017).

3. Glynos C. *et al.* The role of soluble guanylyl cyclase in chronic obstructive pulmonary disease. *Am J Respir Crit Care Med*. **188**, 788-799 (2013).
