# Supplementary material for: NF-κB-responsive miR-155 induces functional impairment of vascular smooth muscle cells by downregulating soluble guanylyl cyclase
Source: Exp Mol Med. 2019 Feb 15;51(2):17. doi: 10.1038/s12276-019-0212-8 (PMC6376011; doi:10.1038/s12276-019-0212-8)
Supplement: Supplementary file 2 — Supplemental Figures [file 12276_2019_212_MOESM2_ESM.pdf]

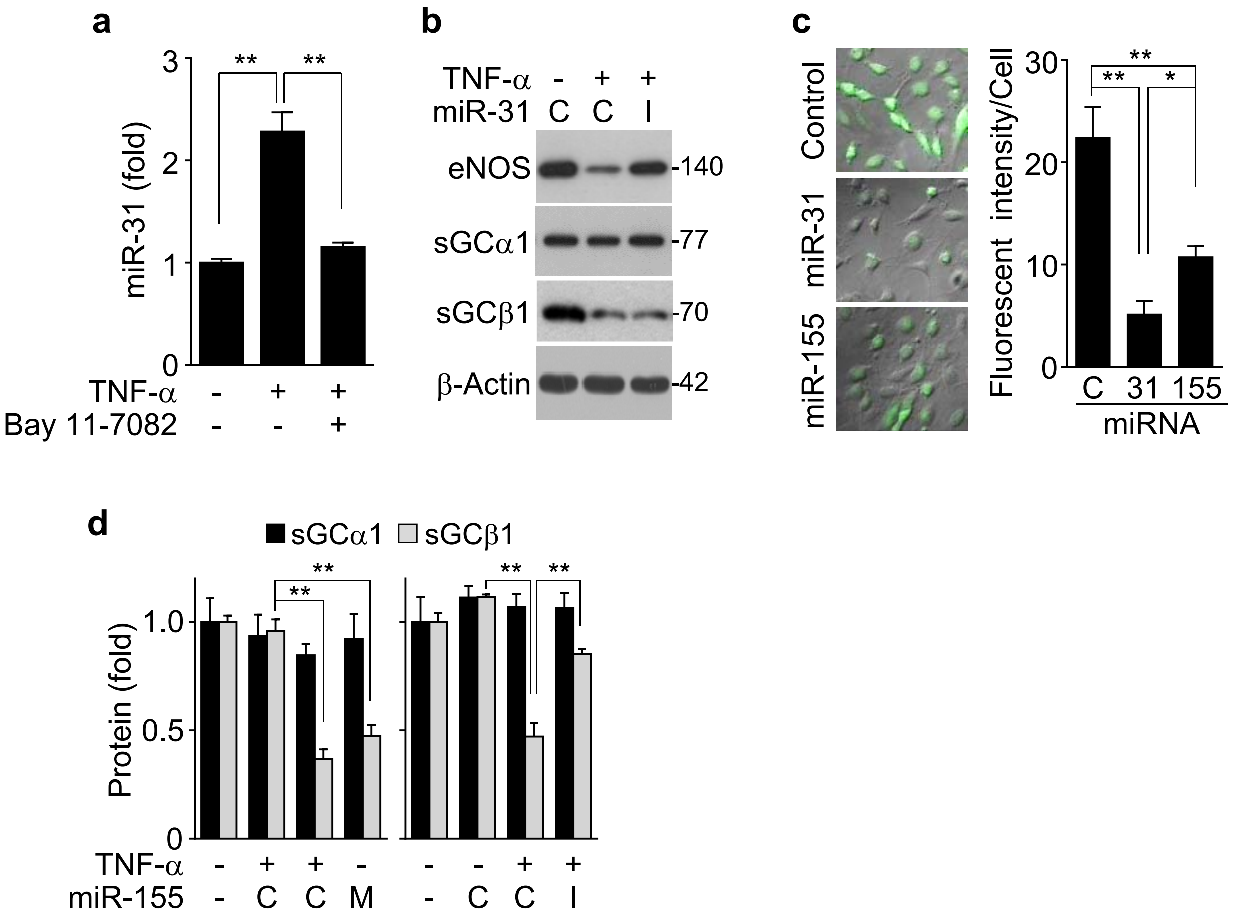

Supplementary Figure 1. TNF-α induces miR-31 which inhibits eNOS expression. (**a**) HUVECs were stimulated with TNF-α (10 ng/mL) in the presence or absence of an NF-κB inhibitor (Bay 11-7082, 5 μM) for 24 h. MiR-31-5p levels were determined by qRT-PCR (n=3). (**b**) HUVECs were transfected with 80 nM of control for miRNA inhibitor (C) or miR-31 inhibitor (I) and treated with or without TNF-α (10 ng/mL) for 24 h. Protein levels of eNOS, sGCα1, or sGCβ1 were determined by Western blotting and quantified using ImageJ software. (**c**) Intracellular NO levels were determined by confocal microscopy using DAF-FM. (n=4). (**d**) HUVECs were transfected with 80 nM of specific control (C) for miR-155 mimic or miR-155 inhibitor, miR-155 mimic (M), or miR-155 inhibitor (I) and stimulated with or without TNF-α (10 ng/mL) for 24 h, protein levels of sGCα1 and sGCβ1 were measured by Western blotting and quantified using ImageJ software (n=3). *P<0.05 and **P<0.01.

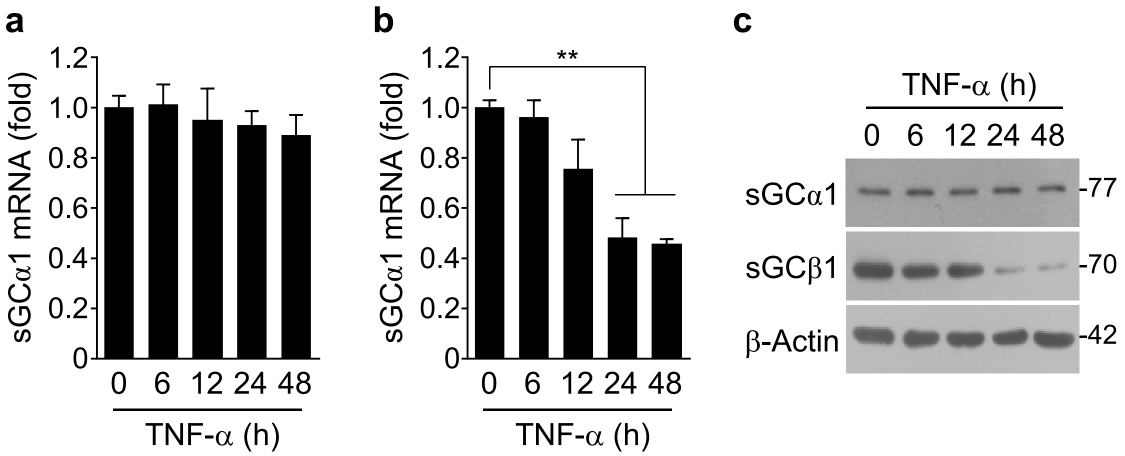

Supplementary Figure 2. TNF-α inhibits the expression of sGCβ1, but not sGCα1, in VSMCs. (**a-c**) HASMCs were treated with TNF-α (10 ng/mL) for the indicated time periods. sGCα1 and sGCβ1 mRNA and protein levels were measured by qRT-PCR (n=3) and Western blotting, respectively. $^{**}P<0.01$.

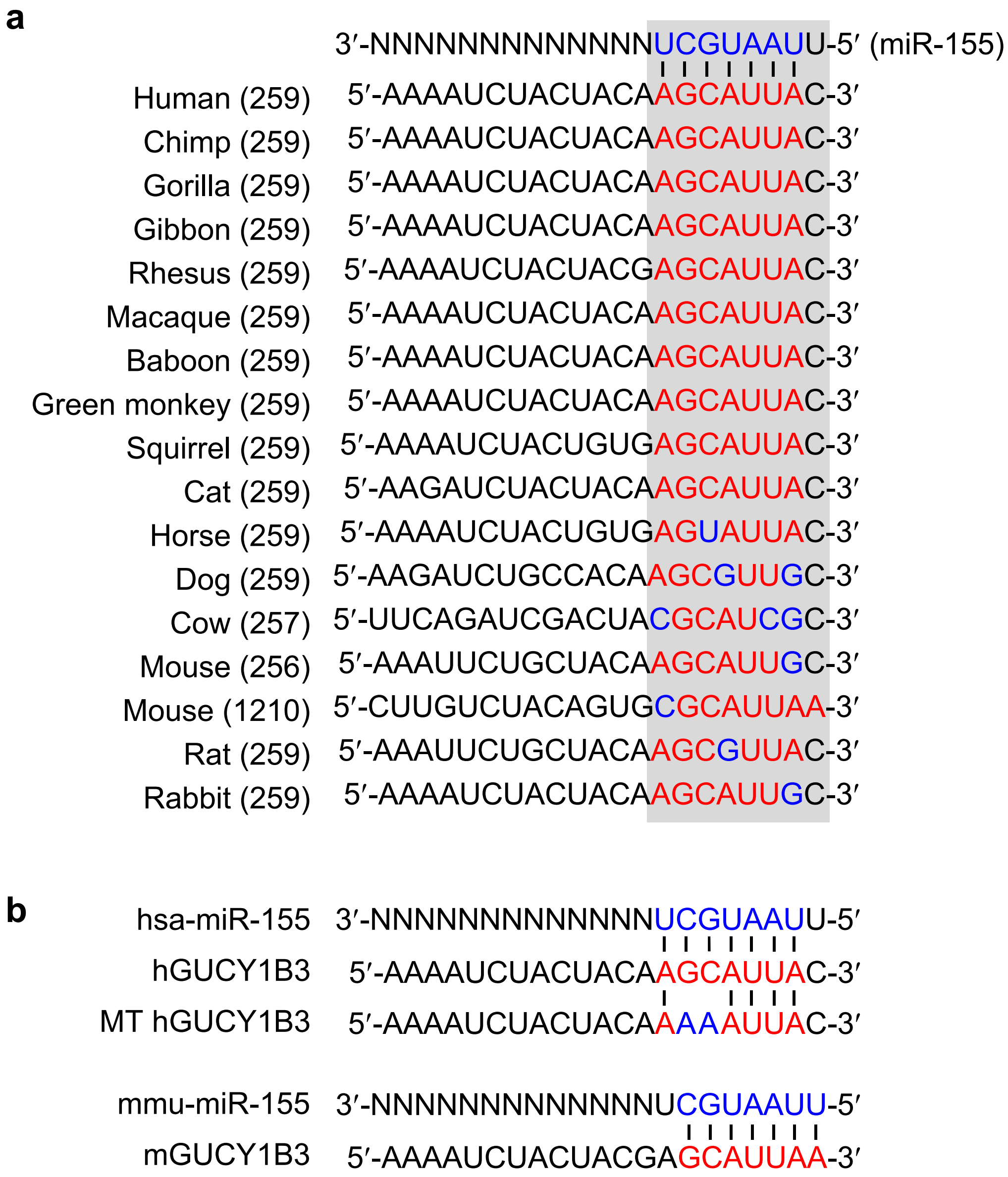

Supplementary Figure 3. Sequence alignment of miR-155 with 3′-UTR of sGCβ1 mRNA in various animals. (**a**) Computational analysis of putative miR-155 complementary sequence in the sGCβ1 mRNA 3′-UTRs in human, non-human primates, and other species using TargetScan, microRNA.org, and miRDB. (**b**) Predicted miR-155 target sequences in the 3′-UTRs of human and mouse sGCβ1 mRNAs, and mutation (MT) of the target site in the human sGCβ1 mRNA 3′-UTR.

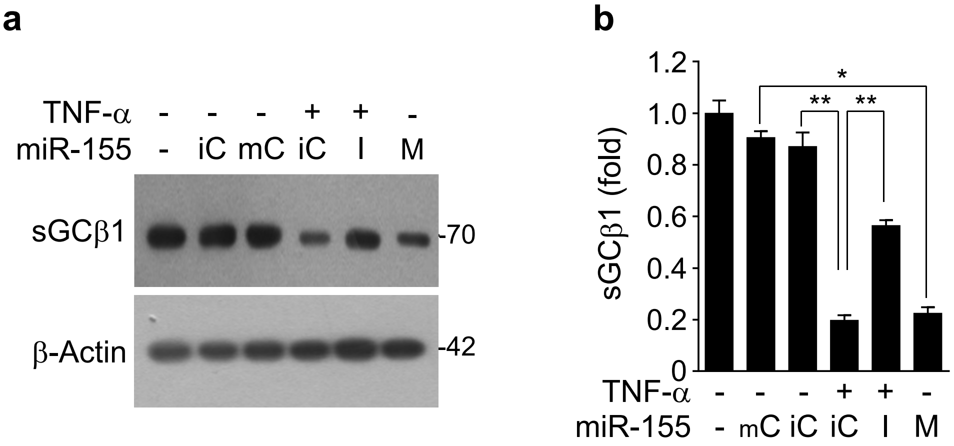

Supplementary Figure 4. TNF-α-induced miR-155 regulates sGCβ1 protein expression in VSMCs. HASMCs were transfected with 80 nM of negative controls for miR-155 mimic (mC) and miR-155 inhibitor (iC), miR-155 inhibitor (I), or miR-155 mimic (M) and stimulated with or without TNF-α (10 ng/mL) for 24 h. sGCβ1 protein was determined by Western blotting. (**a-b**) sGCβ1 were measured by Western blotting and quantified using ImageJ software (n=3). The negative controls (mC and iC) did not alter the basal expression level of sGCβ1. Thus, mC was used as a common negative control for both miR-155 mimic and inhibitor in further experiments. *$P<0.05$ and **$P<0.01$.

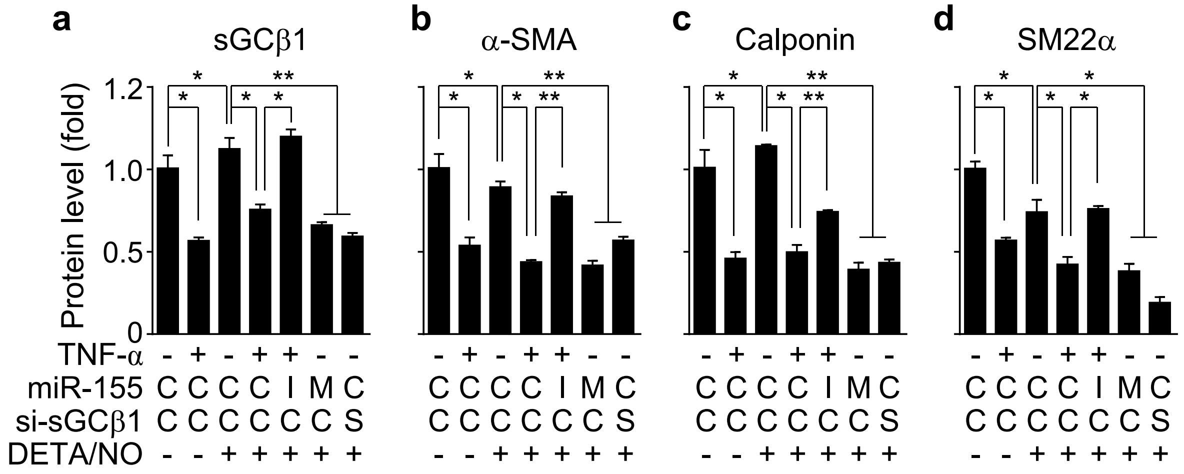

Supplementary Figure 5. TNF-α-induced miR-155 negatively regulates protein expression of VSMC-specific marker genes. HASMCs were transfected with 80 nM of control for miRNA or siRNA (C), miR-155 inhibitor (I), miR-155 mimic (M), or sGCβ1 siRNA (si- sGCβ1), followed by treatment with or without TNF-α (10 ng/mL) for 24 h. (**a-d**) Protein levels of VSMC-specific marker genes were determined by Western blotting and quantified using ImageJ software (n=3). *P<0.05 and **P<0.01.

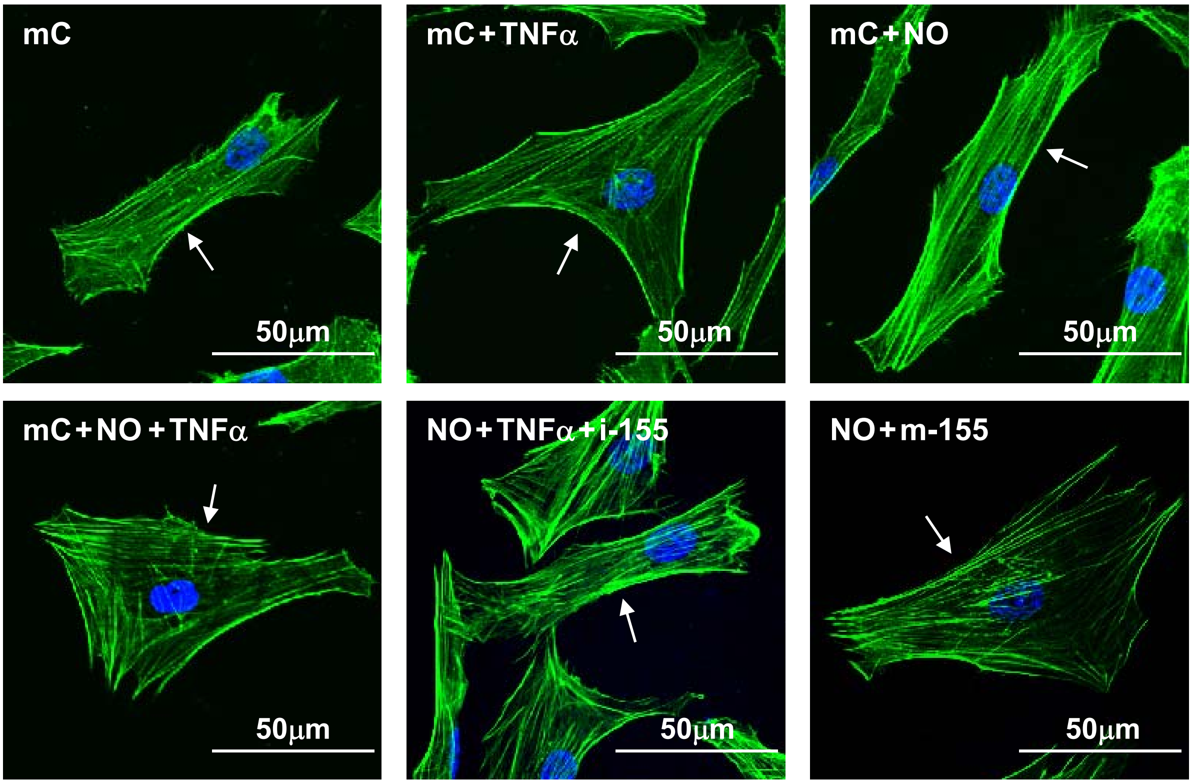

Supplementary Figure 6. TNF-α-induced miR-155 induces morphological alterations in cultured VSMCs. HASMCs were transfected with 80 nM of control miRNA (mC), miR-155 mimic (m-155), or miR-155 inhibitor (i-155) and stimulated with or without TNF-α (10 ng/mL) for 24 h, followed by treatment with DETA/NO (NO, 100 μM) for another 24 h. Representative confocal images were obtained after staining actin filaments with FITC-labelled phalloidin, and nuclei were stained with DAPI. Arrows indicate typical cells with morphological alteration.

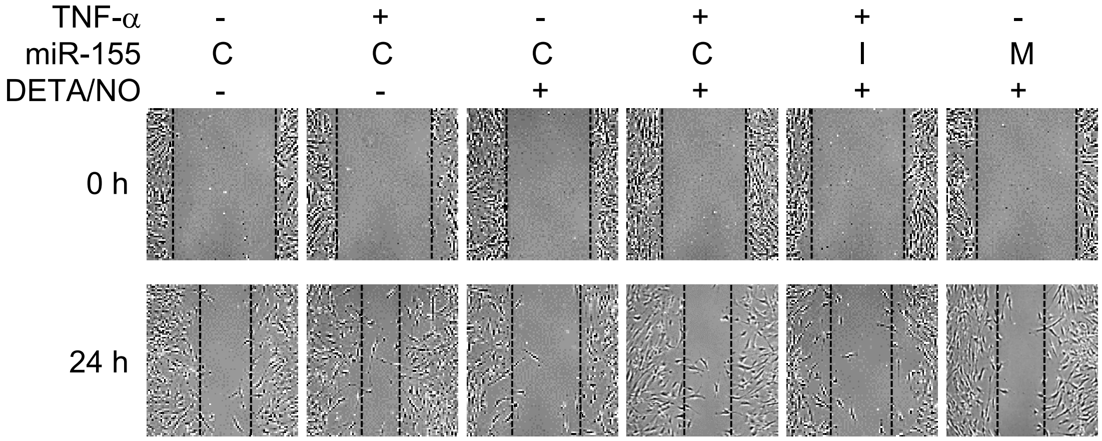

Supplementary Figure 7. TNF-α-induced miR-155 stimulates VSMC migration. HASMCs were transfected with 80 nM of control miRNA (C), miR-155 mimic (M), or miR-155 inhibitor (I) and stimulated with TNF-α (10 ng/mL) for 24 h, followed by treatment with or without DETA/NO (100 μM) for another 24 h. Cell migration was assessed using a microscope equipped with a digital camera.

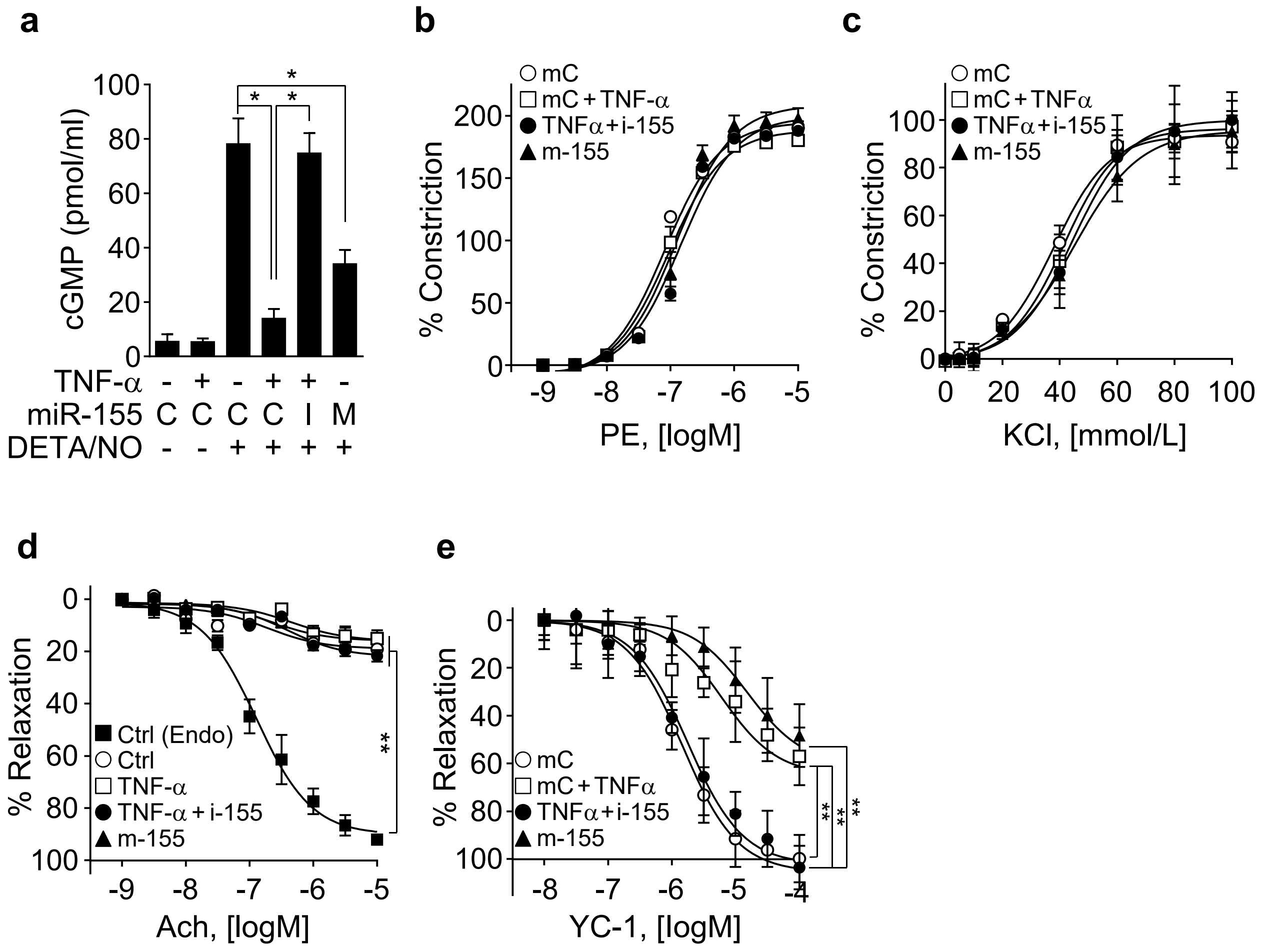

Supplementary Figure 8. TNF-α-induced miR-155 inhibits sGC-dependent vasorelaxation. Mouse de-endothelialized aortic rings were transfected with 100 nM of control miRNA (C), miR-155 (M, m-155), or miR-155 inhibitor (I, i-155), followed by stimulation with or without TNF-α (20 ng/mL) for 24 h. (**a**) Pretreated aortic rings were exposed to DETA/NO (100 μM) for another 24 h. cGMP levels were determined in culture media using a cGMP assay kit (n=3). (**b-e**) Cumulative vascular constriction and relaxation responses of the pretreated de-endothelialized aortic rings to phenylephrine (PE, n=7), acetylcholine (Ach, n=8), KCl (n=5), or the sGC activator YC-1 (n=5) were measured by strain-gauge plethysmography. Intact or endothelialized aortic rings (Endo. shown in **d**) were used as positive controls. *$P<0.05$ and **$P<0.01$.

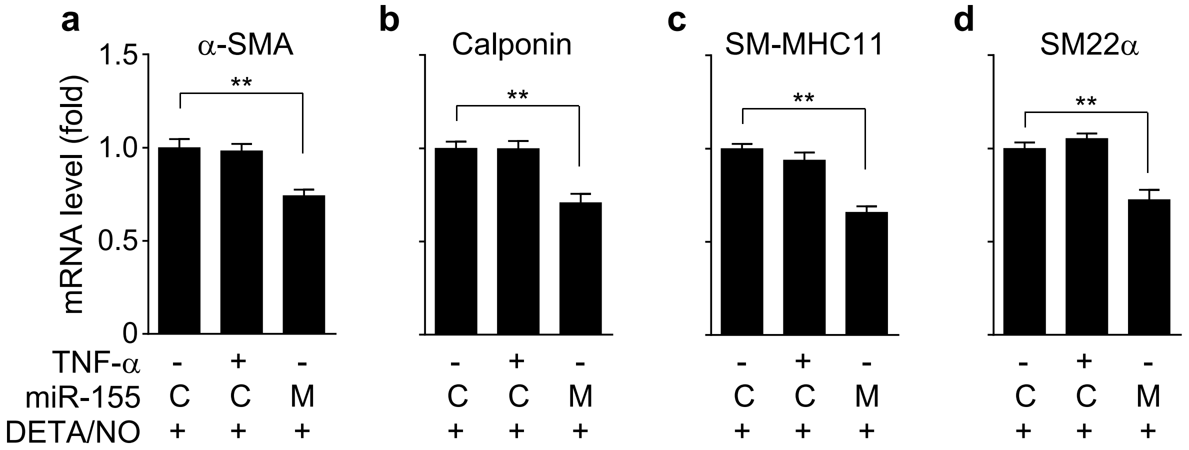

Supplementary Figure 9. TNF-α does not induce VSMC-specific gene expression in miR-155$^{-/-}$ aortic vessels. De-endothelialized aortic rings from miR-155$^{-/-}$ (KO) mice were transfected with 100 nM of control miRNA (C) or miR-155 mimic (M) and stimulated with or without TNF-α (20 ng/mL) for 24 h, followed by treatment with DETA/NO (100 nM) for another 24 h. (**a-d**) VSMC-specific phenotype marker genes were analyzed by qRT-PCR (n=5). ***P*<0.01.

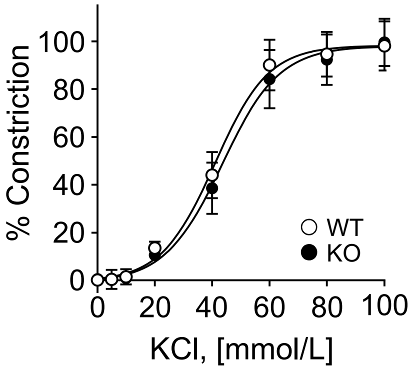

Supplementary Figure 10. KCl-induced vasoconstriction showed similar responses compared to wild-type from miR-155$^{-/-}$ mice vessels. Cumulative vascular constriction responses of de-endothelialized wild-type (WT) and miR-155$^{-/-}$ (KO) aortic rings to KCl were measured by strain-gauge plethysmography (n=5).

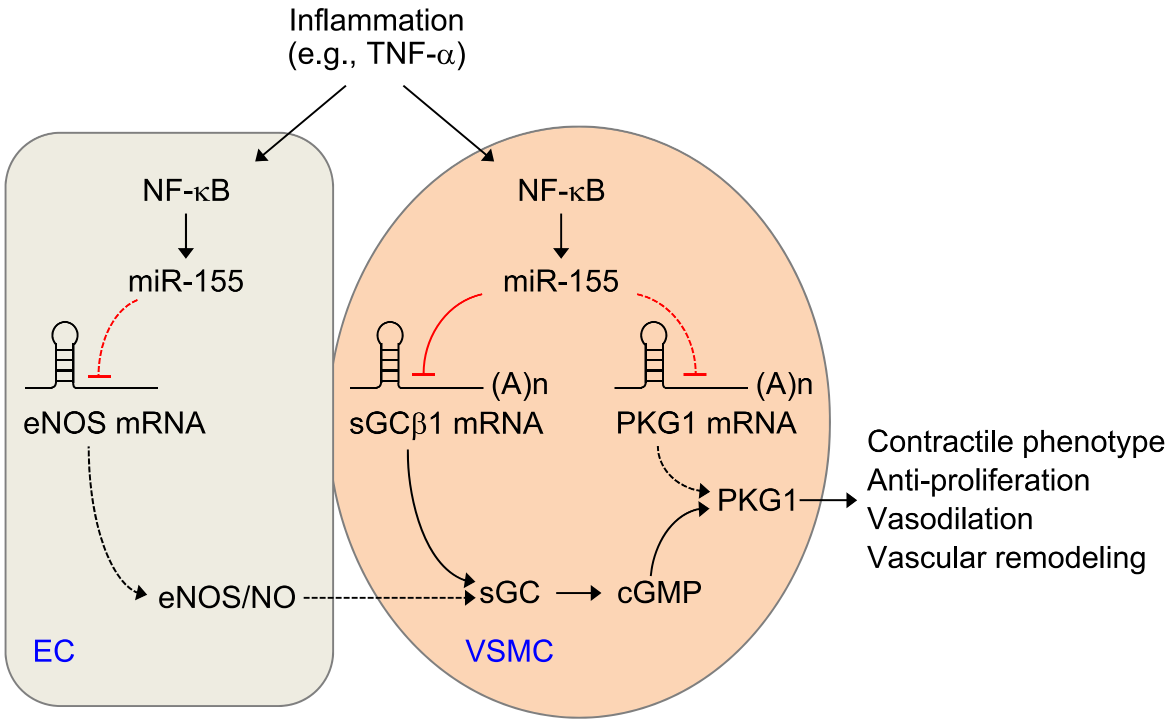

Supplementary Figure 11. Schematic illustration of inflammation-mediated vascular dysfunction. Inflammatory cytokines including TNF-α increase NF-κB-responsive miR-155 in endothelial cells (EC) and VSMCs. MiR-155 inhibits the eNOS/NO pathway in ECs by targeting the 3′-UTR of eNOS mRNA, as well as suppresses the sGCβ1/cGMP/PKG1 axis by targeting the 3′-UTRs of sGCβ1 and PKG1 transcripts, leading to impairment of vascular function. These events contribute to the pathogenesis of inflammation-associated vascular disorders, such an atherosclerosis and preeclampsia, by impairing the VSMC contractile phenotype, vascular remodeling, and vasodilation. The dashed lines indicate the possible regulatory pathways modified from previous data[14,15,21,47].
